# Removal of particulate matter and trace elements from ambient air by urban greenery in the winter season

**DOI:** 10.1007/s11356-018-3628-0

**Published:** 2018-11-07

**Authors:** Arkadiusz Przybysz, Gayane Nersisyan, Stanisław Waldemar Gawroński

**Affiliations:** 1 Laboratory of Basic Research in Horticulture, Faculty of Horticulture, Biotechnology and Landscape Architecture, Warsaw University of Life Sciences – SGGW, Nowoursynowska 159, 02-776 Warsaw, Poland; 2 Biochemistry Department of the Center for Ecological-Nooshere Studies of the National Academy of Sciences of the Republic of Armenia, RA, Yerevan,0025, Abovyan 68 str, Yerevan, Armenia

**Keywords:** Air quality, Evergreen trees and shrubs, Heavy metals, In-wax PM, Phytoremediation, PM size fractions, Surface PM

## Abstract

Particulate matter (PM) is one of the most harmful inhaled pollutants. When PM is emitted into the atmosphere, the only possible method for cleaning ambient air is through vegetation acting as biological filters for pollutants. However, in winter periods when the concentration of PM is usually the highest, the efficiency of plants is very low. The aim of this work was therefore to examine the accumulation of PM and selected trace elements (TE) by three species, evergreen coniferous *Taxus baccata* L. and *Pinus nigra* Arn., and deciduous *Carpinus betulus* L. during the winter season. The highest amounts of PM accumulated on the foliage of *P. nigra*, while TE on the leaves of *C. betulus*. Most of the PM accumulated on plant foliage belonged to the large fraction size (10–100 μm) and was deposited on the surface of foliage (_S_PM). The concentration of four TE (Ni, Pb, Cd, and Sb) was higher in PM accumulated on foliage, while in the case of three other TE (Zn, Cr and Mg), their concentration was higher in plant tissue. The TE were recorded in all PM size fractions and were rather equally distributed between surface PM (_S_PM) and in-wax PM (_W_PM). These findings have implications for urban plantings in countries with short vegetative season, where tolerant conifer species and deciduous species which keep foliage through winter should be included in urban forest plantings due to their efficiency in the removal of pollutants from the air.

## Introduction

### PM in ambient air in winter

In urban areas, air pollution is an increasing threat to human health (EEA 2015). One of the most dangerous inhaled pollutants is particulate matter (PM) (Kim et al. 2015), which is composed of liquid and solid particles, both organic and inorganic (Bell et al. 2011), with an aerodynamic diameter in the range of 0.001–100 μm (Farmer 2002). Chronic exposure to PM is the cause of various health problems (Kim et al. 2015).

PM comes from a wide range of natural and anthropogenic sources (Juda-Rezler et al. 2011). The concentration of PM in ambient air is especially high during winter periods and cold episodes, when PM emission is increased by greater car traffic and the necessity of domestic heating (Majewski et al. 2011). During winter, the unfavourable synoptic-scale (anti-cyclonic circulation) and local meteorological conditions (very low temperature, low wind speeds, surface layer inversions) additionally contribute to the occurrence of increased air pollution events (Juda-Rezler et al. 2011). Moreover, PM emitted from car exhaust systems and individual heating during winter months contains many toxic compounds, including trace elements (TE) and organic pollutants, such as polycyclic aromatic hydrocarbons (PAHs) (Dimitriou and Kassomenos 2017).

### PM removal from ambient air in winter

If PM has been released into the atmosphere, the only possible way to remove it from the ambient air is via vegetation. Sæbø et al. (2012) and Popek et al. (2013) demonstrated the ability of many deciduous trees and shrubs to accumulate PM during or at the end of the growing season. Unfortunately, in the temperate climate, most of these plants shed leaves for winter. Therefore, in countries where the growing season is short and high concentrations of PM are emitted in the winter season, evergreen species could be a more suitable choice for urban plantings. In contrast to deciduous plants, the foliage of evergreen plants is available for PM deposition all year round. The efficiency of evergreen species in PM accumulation has been described in several studies, but also, in this case, most experiments were performed during or just after vegetative season (Beckett et al. 2000; Freer-Smith et al. 2005; Mori et al. 2015; Przybysz et al. 2014b; Sæbø et al. 2012).

Among the evergreens, coniferous plants are an excellent choice for air purification due to the abundant wax layer on the needles, smaller leaves, and more complex shoot structures (Freer-Smith et al. 2005). These features, along with conditions causing high air turbulence inside the tree crowns, contribute to higher interception capacity of contaminants by coniferous plants (Bunzl et al. 1989). However, these species usually keep needles for more than 1 year and thus become so efficient in the retention of pollutants that they die because of too ‘heavy loads’ of contaminants (Gawroński et al. 2017). Therefore, conifers from generas tolerant to air pollutants should be selected (e.g. *Taxus baccata*) or more sensitive species should be planted at an adequate distance from the emission source (Sæbø et al. 2012). Another solution may be the use of evergreen broad-leaved plants (e.g. *Hedera helix*) or plants which keep last year foliage through the winter period (e.g. *Carpinus betulus*).

### TE removal from ambient air

The chemical composition of PM is diverse and consists of more than 40 chemical components, including TE (WHO 2013). Anthropogenic activities (i.e. industry, domestic heating, transport) lead to the contamination of the atmosphere with TE (Shahid et al. 2017; Soleimani et al. 2018). After emission, TE particles are is very mobile in the air (Shahid et al. 2017) and may easily attach with PM (Eqani et al. 2016) due to their integration into the matrix structure during the incineration process or adsorption on to the surface of ferri-magnetics PM found in the atmosphere (Norouzi et al. 2016). A considerable quantity of atmospheric TE is absorbed via foliar organs of plants after the wet or dry deposition of atmospheric fallouts on plant canopy (Shahid et al. 2017). Uzu et al. (2010) showed that not all airborne TE is immobilised on the foliage surface, some of TE linked to PM can enter inside plant leaf tissues. Therefore, the concentrations of TE in the foliage of plants growing close to the emission source are usually high (Mori et al. 2015; Przybysz et al. 2014b; Sæbø et al. 2012). However, it is difficult to state exactly what amount of TE was taken up from the soil and was translocated to the aboveground parts of plants or came from the increased deposition on the foliage (Sæbø et al. 2015).

The aim of this work is therefore to document the accumulation of PM and TE by three species: *Taxus baccata* L., *Pinus nigra* Arn. and *Carpinus betulus* L. during the winter season. We tested the following hypotheses: (1) the three species differ in the accumulation of pollutants on the foliage, (2) the coniferous species accumulate more PM and TE than broad-leaved *C. betulus*, and (3) the concentrations of TE in PM accumulated on the foliage are higher than in the plant tissue.

## Material and methods

### Plant material and experimental locations

The objects of this study were three tree species: Common yew (*Taxus baccata* L.), Black pine (*Pinus nigra* L.) and Common hornbeam (*Carpinus betulus* L.). Plants were grown in the campus of Warsaw University of Life Sciences – SGGW. The plants were in good condition (healthy and free from pests), and had already been growing in the studied location for several years. The study site was moderately polluted, located in the suburban area of Warsaw, but with relatively heavy traffic resulting from the large number of students’ cars and public transport during the winter period. For the determination of PM and TE quantity on the foliage, the plant material was collected at the beginning of February 2017. The daily mean level of PM10, PM2.5 in the air and meteorological data (temperature and precipitation) for 30 days before sampling are presented in Table 1. The samples were harvested from four plants (biological replications), and always from the traffic-exposed side of the plant, at 1.5–1.7 m above the ground level (the height of the human face). Each sample was composed of 2–3 shoots of yew or pine and 10–15 Common hornbeam leaves, and was 300–500 cm^2^ large. After harvesting, the samples were placed in paper bags and stored (22 °C, 70% RH) for about 1 week before analysis, which prevented them from rotting and allowed all samples to be analysed as air dried material.

**Table 1:** The daily average air concentrations of PM10 and PM2.5, total precipitation and daily average temperature in the study site for 30 days before sampling

Parameter	Date																																		
	01.01	02.01	03.01	04.01	05.01	06.01	07.01	08.01	09.01	10.01	11.01	12.01	13.01	14.01	15.01	16.01	17.01	18.01	19.01	20.01	21.01	22.01	23.01	24.01	25.01	26.01	27.01	28.01	29.01	30.01	31.01	01.02	02.02	03.02	04.02
PM 2.5^1^ (μg m^−3^)	30.4	30.9	20.9	9.32	8.70	12.0	39.0	89.6	86.6	66.1	53.7	30.9	24.0	24.1	31.7	38.0	65.8	55.0	31.8	32.2	26.7	23.5	41.2	65.9	32.2	33.0	66.3	87.3	39.4	43.6	51.3	53.2	61.5	65.9	29.6
PM 10^1^ (μg m^−3^)	41.2	38.4	26.3	11.2	12.5	20.8	59.8	116.1	126.3	80.3	70.6	37.9	47.8	31.9	42.3	51.5	88.5	65.3	41.3	48.8	33.2	30.2	58.3	88.8	38.2	50.5	98.7	107.8	54.5	57.8	70.9	72.0	87.6	80.0	37.3
Precipitatipn^2^ (mm)		0.8	7.1	5.4								1.6	1.6		1.2	0.2			0.1	0.2				0.1									0.9		
Temperature^2^ (°C)	1.5	0.9	− 0.7	1.2	− 7.1	− 13.6	− 15.6	− 12.9	− 9.5	− 8.3	− 9.1	− 0.6	0.2	0.6	− 1.0	− 1.0	− 2.4	− 6.9	− 5.8	− 1.7	1.0	0.2	− 0.7	− 2.6	0.3	− 4.0	− 4.4	− 3.0	− 1.7	− 2.9	− 3.6	− 3.5	− 1.0	− 0.2	− 2.8

^1^Data obtained from the Chief Inspectorate of Environmental Protection

^2^Data obtained from the Polish Inspectorate of Environmental Protection and Polish Weather Services

In order to determine the concentration of selected TE in soil as well as soil pH and electroconductivity, the pooled soil samples (8 individual samples) were collected from the root zone of the examined plants at the end of October 2016. Before collecting the samples, plant cover and the upper 1–3 cm of topsoil were removed. The soil that came into immediate contact with the metal spade was removed, and the final soil samples were collected with a wooden spoon. Before measurement, soil samples were dried in Laboratory Convection Oven (SANYO MOV-212F Dry Heat Sterilizer) at 75 °C for 72 h. The concertation of TE was determined ex situ with XRF (Innov-X system®, alpha-4000 series) equipped with an X-ray tube as an irradiation source and calibrated with the Compton normalization method. The results are presented in Table 2.

**Table 2:** Concentration of selected elements in the soil as well as pH and EC of soil at the study sites. Data are means ± SD, *n* = 8

Ba	Ca	Cd	Cu	Cr	Fe	Mn	Ni	Pb	Sb	Zn	EC (μS cm^−1^)	pH
mg kg^−1^ DW												
234.2 ± 16.1	7427 ± 595.4	11.7 ± 3.62	21.6 ± 0.61	33.2 ± 1.42	8865 ± 143.1	243.3 ± 5.04	nd^1^	23.1 ±1.77	29.4 4.65	50.4 ± 8.45	1406 ± 42.8	7.18 ± 0.04

^1^Concentration of Ni was below the detection level

### Quantitative assessment of PM and leaf wax content

The content of PM was examined according to Dzierżanowski et al. (2011). Two categories of PM: (i) water-washable from leaf surfaces (_S_PM) and (ii) that retained in leaf wax (_W_PM) were determined. The plant material was first washed for 60 s with 250 mL distilled water and thereafter for 40 s with 150 mL chloroform. The fractional division for both categories was done sequentially. The washing solutions were first sieved through a metal sieve (retention 100 μm, Haver & Boecker, Germany) and then filtered through a 10-μm paper filter (Whatman, UK, Type 91), then a 2.5-μm paper filter (Whatman, UK, Type 42) and finally a 0.2-μm PTFE membrane filter (Whatman, UK). The filtration was carried out using a filtration set equipped with 47-mm glass filter funnel (PALL Corp., USA) connected to a vacuum pump. Three fractions of PM were thus collected: (i) 10–100 (large), (ii) 2.5–10 (coarse) and (iii) 0.2–2.5 μm (fine). The sum of all PM fractions was designated as total PM. The filters were dried for 45 min at 60 °C, stabilised in the weighing room for 45 min and weighed before and after filtration (balance XS105DU, Mettler-Toledo International Inc. and deioniser gate, HAUG, both Switzerland). The amount of waxes dissolved in chloroform was assayed for every plant sample in pre-weighed beakers after chloroform evaporation. Total leaf area of plant samples was measured (Image Analysis System, Skye Instruments Ltd., UK and Skye-Leaf software), allowing the amount of PM and waxes to be always expressed in this work as micrograms per square centimeter.

### Quantitative assessment of TE

The determination of the selected TE (Zn, Mg, Pb, Mn, Ni, Cr, Pt, Cd and Sb) was performed on the material (washed plant samples and filters with collected PM) obtained after the quantitative assessment of PM (2.2.). The concentrations of TE were measured through the atomic-absorption method (AAnalyst 800, Perkin Elmer, USA) consistent with ISO-8288, ISO-5666 and ISO-11696 standards.

In order to determine the TE concentration in plant tissue, the dry ashing method was applied. After analysis of PM accumulation, the samples were stored in paper bags until they were washed with distilled water (MILIQ), chopped into small pieces, air dried at room temperature and weighed accurately. The subsamples of 0.2–2.0 g (depending on the expected concentrations of the elements to be determined) of the dry plant material were ground in a laboratory mill and 0.5–1 g of ground dried plant sample was placed in a porcelain crucible. Then, the porcelain crucible was placed in a cool muffle furnace and the samples were ashed at 500 °C overnight. The ash residues were cooled down to room temperature and dissolved in 5 mL of 20% HCl. The obtained solution was filtered through acid-washed filter paper into a 50-mL volumetric flask, diluted with distilled water (MILIQ) and mixed.

To assess the TE concertation in PM accumulated on the foliage, filter samples were wet digested. The amount of PM accumulated on a single filter was not sufficient enough; thus, the analyses were performed in two replicates, where a single replicate consists of two filters with PM from the same species, category and size fraction. The filters with accumulated PM were placed in a small beaker and 10 mL of concentrated HNO_3_ was added. The samples were incubated overnight. Then, samples were heated carefully on a hot plate until the production of red NO_2_ fumes had ceased. After cooling down the beaker, a small amount (2–4 mL) of 70% HClO_4_ was added. The solution was heated again and allowed to evaporate to a small volume. Each sample was transferred to a 50-mL volumetric flask, diluted to volume with distilled water (MILIQ) and mixed.

### Statistics

The data were subjected to one-factorial analysis of variance using Statgraphics Plus 4.1 (Statpoint Technologies Inc., Warrenton, VA, USA). The differences between means of combinations were evaluated by post hoc Tukey’s honestly significant difference (HSD) test. The means were considered to be significantly different at *P* < 0.05. The data are presented as mean ± SD. The correlations between the amount of ambient air PM and meteorological conditions were calculated using Pearson’s correlation coefficient in Microsoft Excel.

## Results

### Effect of meteorological conditions on ambient air PM concentration

The air PM concentration in January 2017 often exceeds the permitted EU limits, which are 40 μg m^−3^ (yearly average) and 50 μg m^−3^ (24-h average) for PM10, and 25 μg m^−3^ (yearly average) for PM2.5 (Table 1). The concentration of PM (both PM10 and PM2.5) in the air was slightly negatively correlated with ambient temperature recorded during the 30 days before sampling (*r* = − 0.312 and *r* = − 0.340 for PM2.5 and PM10 respectively). A weak relationship between PM air concentration and temperature may be explained by the fact that the increase of PM concentration resulted most probably from individual heating which is always delayed by 1 or 2 days compared to the fall of temperature. January 2017 was rather dry and only intensive precipitation (recorded between 3rd and 4th January) decreased the air PM concentration (Table 1).

### Quantitative assessment of PM and leaf wax content on plant foliage

**Fig. 1:**
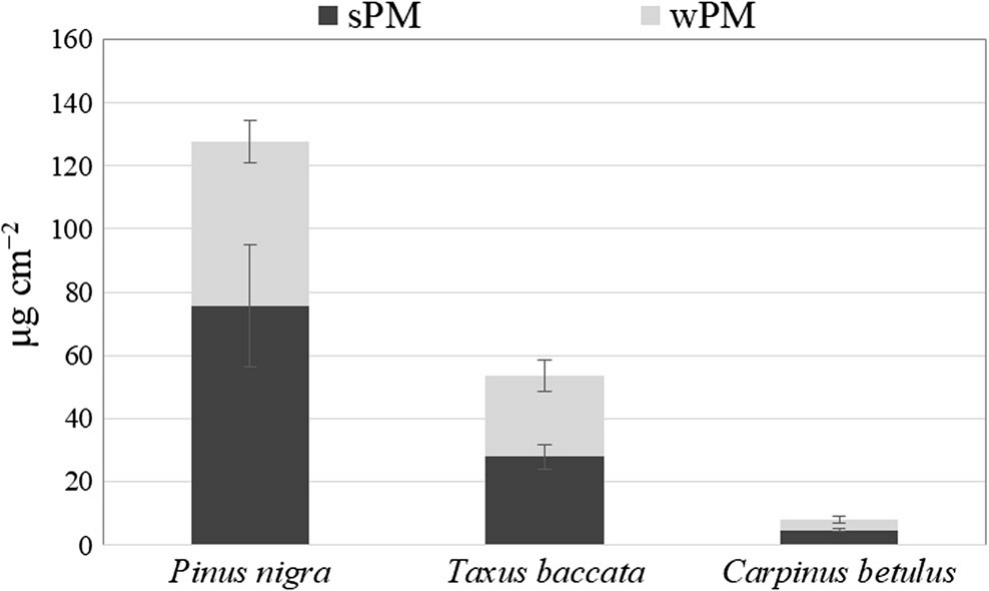
The amount (μg cm^−2^) of total PM, surface PM (_S_PM) and wax-embedded PM (_W_PM) accumulated on the foliage of three tested species. Data are mean ± SD, *n* = 4. ^1^Comparison of the total PM amount between different species (capital letters). ^2^Comparison of the surface PM (_S_PM) and wax-embedded PM (_W_PM) within the same species (lowercase letters)

The total PM accumulation differed significantly between species (Fig. 1). Among the species tested, the black pine had the greatest mass of total accumulated PM, while the common hornbeam had the lowest (15.7 times less than pine). The plants accumulated PM both on foliage surfaces (_S_PM) and in waxes (_W_PM). With regard to quantity, _S_PM exceeded _W_PM and contributed to 52% (yew) or 60% (black pine and common hornbeam) of the total PM accumulation (Fig. 1).

The black pine plants accumulated significantly the greatest mass of PM in all size fractions (Table 3). On all species, the largest size fraction (10–100 μm) made up significantly the greatest proportion of accumulated PM mass and the fine fraction made up the smallest proportion. Expressed as percentage, the large PM fraction amounted to 90% in *P. nigra* and *T. baccata*, and 49% in *C. betulus*, while fine PM accounted for only 3% in *P. nigra* and *T. baccata* or 16% in *C. betulus*. Regardless of the species, the amount of large PM was higher on the foliage surface (_S_PM) than in waxes (_W_PM), but significantly so only in the case of large PM accumulated by the black pine. The amount of coarse and fine PM did not differ between the two PM categories (Table 3).

**Table 3:** Total amount and division into categories (sPM and wPM) of large (10–100 μm), coarse (2.5–10 μm) and fine (0.2–2.5 μm) PM accumulated on foliage of examined plant species. Data are means ± SD, *n* = 4

Species	PM (μg cm^−2^) Size fraction (μm)			10–100 μm (μg cm^−2^) PM category (surface/in waxes)		2.5–10 μm (μg cm^−2^) PM category (surface/in waxes)		0.2–2.5 μm (μg cm^−2^) PM category (surface/in waxes)	
	10–100	2.5–10	0.2–2.5	sPM	wPM	sPM	wPM	sPM	wPM
*Pinus nigra*	114.6 ± 15.7 A^1^ (a^2^)	8.99 ± 2.59 B (a)	4.13 ± 0.74 B (a)	70.2 ± 17.0 A (a)	44.3 ± 4.74 B (a)	3.63 ± 3.15 A (a)	5.36 ± 1.03 A (a)	1.84 ± 0.71 A (a)	2.30 ± 1.41 A (a)
*Taxus baccata*	48.4 ± 3.26 A (b)	3.77 ± 0.58 B (b)	1.45 ± 0.53 B (b)	25.9 ± 3.91 A (b)	22.5 ± 5.10 A (b)	1.47 ± 0.45 A (a)	2.67 ± 0.98 A (b)	0.81 ± 0.23 A (b)	0.64 ± 0.33 A (a)
*Carpinus betulus*	3.97 ± 0.54 A (c)	2.79 ± 0.20 B (b)	1.33 ± 0.70 C (b)	2.34 ± 0.69 A (c)	1.62 ± 0.58 A (c)	1.66 ± 0.73 A (a)	1.13 ± 0.59 A (b)	0.82 ± 0.23 A (b)	0.50 ± 0.62 A (a)

^1^Comparisons between different PM size fraction (10–100, 2.5–10, 0.2–2.5 μm) or category (sPM and wPM) within the same plant species (capital letters)

^2^Comparisons between different plant species within the same PM size fraction (10–100, 2.5–10, 0.2–2.5 μm) or category (sPM and wPM) (lowercase letters)

**Fig. 2:**
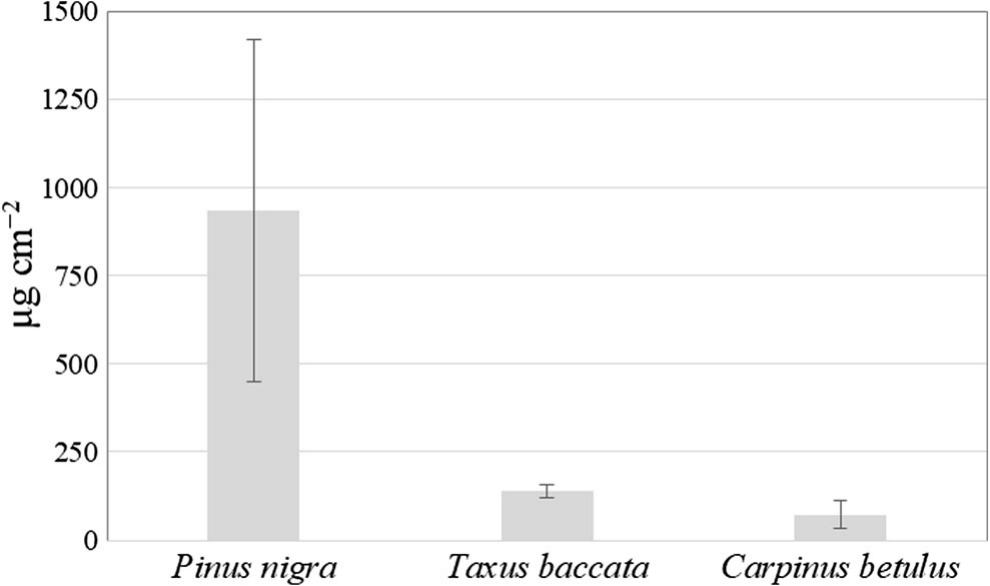
The wax content (μg cm^−2^) in the foliage of three tested species. Data are mean ± SD, *n* = 4

The significantly highest amount of wax was recorded for the black pine and the lowest for the common hornbeam, and the difference between those two species was substantial (12.7 times) (Fig. 2). The wax layer in the yew was only slightly thicker than in the common hornbeam and much thinner than in the black pine (6.7 times) (Fig. 2).

### Quantitative assessment of TE

The three species differed in terms of concentrations of TE in foliage. Higher concentrations, albeit not always significantly, were recorded in *C. betulus* (Table 4). The exception was the high concentration of Sb found in *P. nigra* (Table 4).

**Table 4:** Leaf and PM concentrations (mg kg^−1^ DW) of selected trace elements. Data are means ± SD, *n* = 4 (plant material) or *n* = 2 (PM)

Species	mg kg^−1^ DW						
	In plant	In accumulated PM					
		Total PM	10–100 μm	2.5–10 μm	0.2–2.5 μm	Total sPM	Total wPM
	Zn						
*Pinus nigra*	29.0 ± 5.55 B	36.5 ± 17.3	37.1 ± 19.6	22.5 ± 4.11	54.2 ± 8.63	42.0 ± 28.5	25.2 ± 2.27
*Taxus baccata*	52.9 ± 5.71 A	19.4 ± 5.15	19.7 ± 5.25	18.8 ± 3.42	8.93 ± 4.13	24.3 ± 2.73	14.1 ± 7.07
*Carpinus betulus*	56.5 ± 12.5 A	26.0 ± 1.79	23.5 ± 0.93	18.5 ± 7.43	51.6 ± 12.9	29.4 ± 1.70	21.2 ± 1.59
Average	46.1 ± 4.30	24.1 ± 3.06	26.8 ± 5.00	19.9 ± 1.86	38.2 ± 9.74	31.9 ± 6.20	20.2 ± 2.48
	Mg						
*Pinus nigra*	923.0 ± 318.1 B	268.5 ± 13.5	286.0 ± 12.5	103.0 ± 2.61	142.6 ± 22.9	256.3 ± 36.0	274.1 ± 81.2
*Taxus baccata*	985.4 ± 243.2 AB	107.3 ± 0.25	110.9 ± 1.98	86.2 ± 0.32	51.0 ± 41.7	118.2 ± 8.84	94.9 ± 10.6
*Carpinus betulus*	1632.8 ± 466.1 A	145.6 ± 2.35	116.3 ± 17.9	115.9 ± 4.03	296.5 ± 43.6	126.4 ± 3.74	173.7 ± 13.0
Average	1180.4 ± 134.0	173.8 ± 30.9	171.1 ± 36.6	101.7 ± 5.52	163.4 ± 46.8	166.9 ± 29.1	180.8 ± 36.1
	Ni						
*Pinus nigra*	0.98 ± 0.28 B	3.67 ± 0.00	3.63 ± 0.00	3.17 ± 0.18	5.79 ± 0.27	3.67 ± 0.00	3.66 ± 0.01
*Taxus baccata*	1.01 ± 0.49 B	3.24 ± 0.23	3.33 ± 0.21	2.42 ± 0.13	2.55 ± 0.60	3.06 ± 0.14	3.46 ± 0.64
*Carpinus betulus*	2.24 ± 0.58 A	3.76 ± 0.40	3.49 ± 0.40	3.05 ± 0.01	6.31 ± 1.82	3.73 ± 0.38	3.81 ± 0.42
Average	1.41 ± 0.21	3.55 ± 0.13	3. 48 ± 0.10	2.88 ± 0.15	4.88 ± 0.82	3.48 ± 0.15	3.64 ± 0.15
	Pb						
*Pinus nigra*	1.31 ± 0.59 B	425.7 ± 20.5	390.8 ± 21.0	544.2 ± 32.7	1121.1 ± 103.2	406.8 ± 30.6	456.8 ± 4.47
*Taxus baccata*	1.64 ± 0.55 B	499.2 ± 8.30	519.0 ± 6.48	363.1 ± 35.8	211.1 ± 0.74	538.4 ± 36.7	459.9 ± 51.5
*Carpinus betulus*	3.60 ± 2.35 A	598.2 ± 72.6	439.7 ± 24.1	528.2 ± 12.3	1310.5 ± 556.2	638.3 ± 129.2	542.7 ± 5.53
Average	2.18 ± 0.48	507.7 ± 34.5	449.8 ± 24.3	478.5 ± 37.7	880.9 ± 238.2	527.8 ± 49.3	486.5 ± 20.2
	Cr						
*Pinus nigra*	19.3 ± 4.09 A	12.5 ± 1.20	13.1 ± 1.41	5.67 ± 0.56	9.88 ± 0.85	11.18 ± 2.08	14.2 ± 0.55
*Taxus baccata*	16.6 ± 2.47 A	6.29 ± 0.01	6.54 ± 0.05	4.22 ± 0.21	3.97 ± 0.12	6.32 ± 0.11	6.27 ± 0.14
*Carpinus betulus*	22.5 ± 11.4 A	6.37 ± 0.20	5.32 ± 0.69	5.98 ± 0.67	11.1 ± 3.28	6.67 ± 0.08	5.93 ± 0.62
Average	19.5 ± 2.00	8.38 ± 1.31	8.32 ± 1.55	5.29 ± 0.38	8.32 ± 1.52	8.06 ± 1.06	8.80 ± 1.71
	Cd						
*Pinus nigra*	0.06 ± 0.02 B	80.6 ± 0.40	74.7 ± 0.36	98.3 ± 7.62	205.7 ± 14.4	76.4 ± 1.30	86.5 ± 0.77
*Taxus baccata*	0.04 ± 0.01 B	91.6 ± 1.76	95.8 ± 1.32	63.0 ± 6.52	28.9 ± 1.45	96.0 ± 3.47	87.2 ± 6.80
*Carpinus betulus*	0.11 ± 0.01 A	108.9 ± 6.04	79.0 ± 8.43	100.0 ± 7.96	234.5 ± 83.7	110.2 ± 13.01	107.3 ± 3.93
Average	0.07 ± 0.01	93.7 ± 5.32	83.1 ± 4.36	87.1 ± 7.97	156.4 ± 43.5	94.2 ± 6.67	93.7 ± 4.55
	Sb						
*Pinus nigra*	0.34 ± 0.15 A	6.37 ± 0.56	6.95 ± 0.59	1.53 ± 0.92	0.72 ± 0.21	6.42 ± 0.69	6.26 ± 0.34
*Taxus baccata*	0.15 ± 0.06 A	0.87 ± 0.02	0.94 ± 0.04	0.27 ± 0.09	0.37 ± 0.05	0.73 ± 0.04	1.03 ± 0.02
*Carpinus betulus*	0.15 ± 0.05 A	0.64 ± 0.16	0.77 ± 0.30	0.48 ± 0.10	0.54 ± 0.18	0.78 ± 0.06	0.43 ± 0.31
Average	0.21 ± 0.04	2.63 ± 1.19	2.89 ± 1.29	0.76 ± 0.30	0.54 ± 0.08	2.64 ± 1.20	2.57 ± 1.17

When TE concentrations were investigated in PM accumulated on plant foliage, some trends were identified (Table 4). The species average of four elements (Ni, Pb, Cd and Sb) was higher in the total accumulated PM, while for three other elements (Zn, Cr and Mg), higher concentrations were recorded in plant foliage. The highest concentrations of the tested TE in PM deposited on plant foliage were recorded in *P. nigra* (Zn, Mg, Cr, Sb) or *C. betulus* (Pb, Cd); only concentrations of Ni were similar between species. Regardless of the species, TE were relatively equally distributed between _S_PM and _W_PM. The only exception was Zn which was always recorded in higher concentration in sPM. On average, Zn, Ni, Pb, Cd had the greatest concentrations in PM fraction 0.2–2.5 μm, Sb in 10–100 μm, while for Mg and Cr, clear trends were not recorded (Table 4).

The platinum (Pt) was determined in all samples, but was not recorded in tested plant material and PM deposited on the foliage.

## Discussion

### PM accumulation

PM concentration in ambient air is seasonally dependent, being higher in colder months and usually lower in summer (Majewski et al. 2011). Also, in this work, the concentration of air PM during a month before sampling often exceeded the permitted EU limits and was higher when the temperature decreased below 0 °C, which suggests individual heating and increased traffic as the main sources of airborne PM in the studied area. Urban vegetation has already proven its efficiency in the removal of PM from air in summer (Popek et al. 2013; Sæbø et al. 2012); however, the knowledge of how plants may reduce air pollution level beyond the growing season is still insufficient. Therefore, in this work, three species commonly planted in urbanised areas were tested for their efficiency in PM accumulation on foliage during the winter period. *P. nigra*, *T. baccata* and *C. betulus* showed different capacities in PM accumulation. Of the species tested, *P. nigra* proved to be the most efficient at PM accumulation, while *C. betulus* was the least efficient. These results confirm previous findings that evergreen coniferous plants could be efficient in collecting PM on their foliage (Beckett et al. 2000; Freer-Smith et al. 2005; Mori et al. 2015; Przybysz et al. 2014b; Sæbø et al. 2012). Freer-Smith et al. (2005) showed that conifers capture larger amounts of PM than broad-leaved trees as a result of their aerodynamic properties, small needles and complex shoot structures. The lower PM accumulation on *C. betulus* leaves can be additionally supported by the fact that the examined leaves were developed in the last vegetative season (2016) and their wax layer, which was the lowest among the tested species, was probably also strongly degraded by harsh environmental conditions and the lack of regeneration during autumn and winter months.

In the present study, the level of PM accumulation on the foliage of *P. nigra* and *T. baccata* was considerably greater than that reported in many other studies where similar analytical methods were used (Popek et al. 2013; Sæbø et al. 2012). These findings strongly support the view that the dedicated conifer plantings may be better for local air pollution reduction than deciduous species, because of its potential for a significant remediation effect, especially during winter, when the PM concentration in the air is often highest and poses the greatest threat to human health (Beckett et al. 2000). In all the three tested species, the PM deposited on plant foliage was present both on leaf surfaces (_S_PM) and in waxes (_W_PM), but the quantity of total _S_PM was always higher. A similar relationship has been found by Sæbø et al. (2012) and Popek et al. (2013). In nature, _S_PM can easily be washed off from foliage by precipitation or removed by strong wind, as also pointed out by other authors (Beckett et al. 2000; Przybysz et al. 2014b). Unlike _S_PM, _W_PM is immobilised in the waxes, at least for some time, although wax desquamation can be expected during the lifetime of foliage and _w_PM can be re-suspended into the environment. The pollutants themselves are also capable of degrading wax layer, as it was shown in *Pinus sylvestris* L. (Burkhardt and Pariyar 2014), and may negatively affect plants’ efficiency in further PM immobilisation. For this reason, it seems that during the winter period *C. betulus* may rather serve as a barrier than a filter for contaminated air, while *P. nigra* and *T. baccata* can accumulate and immobilise PM for a longer period of time. However, recorded in this work, relatively large variation in wax content in *P. nigra* needles suggests that winter wax degradation is possible also in conifer species.

Confirming the findings by Popek et al. (2013), Przybysz et al. (2014b) and Sæbø et al. (2012), the large size fraction comprised the largest proportion of all accumulated PM. However, in this study, the proportion of large PM was exceptionally high, up to even 90% in *P. nigra* and *T. baccata*. These data are in agreement with Farmer (2002), who showed that the largest PM fraction is frequently found in polluted areas. Large PM is not as dangerous to human health as PM with a smaller diameter, but it can have a significant negative impact on plants (Przybysz et al. 2014a). It can also be assumed that re-suspension of PM in winter, when the main precipitation is snow, is less likely to happen. According to Przybysz et al. (2014b), large PM are the first to be removed from foliage by rain; thus, such high content of large PM recorded in this work can be explained only by long accumulation, not interrupted by re-suspension events.

We concluded from our results that the potential of evergreen coniferous plants for PM accumulation during winter is high and this property can be used in urban plantings. However, it has to be taken into account, that these plants are at the same time more sensitive to air pollution (Gawroński et al. 2017). Burkhardt and Pariyar (2014) showed that degradation of epicuticular waxes by air pollutants may additionally lead to decreased drought tolerance of *P. sylvestris*. Therefore, conifer species could be used in areas with less than maximum pollution level, but still close to roads, so that direct exposure to de-icing salt and the highest pollution concentrations can be avoided (Sæbø et al. 2012). The selection of most tolerant species/varieties seems to be critical. The species that are very tolerant and well-surviving contamination with PM are *T. baccata* and *P. nigra*. *T. baccata* is characterised with a very efficient self-cleaning mechanism (Gawroński et al. 2017). An interesting alternative might be plantation with deciduous plants which keep the leaves until the next vegetative season, such as *C. betulus* or *Quercus rubra*.

### TE accumulation

It is difficult to state exactly how much of TE is taken up from the soil and or comes from increased air deposition on the foliage (Sæbø et al. 2015); thus, the importance of roadside vegetation in reducing the levels of TE in the ambient air is sometimes underestimated. In this work, TE was analysed in both plant tissues and PM accumulated on the foliage, which allowed to prove that in parallel with the adsorption of PM, plant foliage accumulated also considerable quantities of airborne TE deposited as PM. The concentrations of four TE (Ni, Pb, Cd and Sb) were higher in the accumulated PM, while for three other (Zn, Cr and Mg), higher concentrations were recorded in plant tissues. The TE recorded in higher concentration in PM are associated with anthropogenic sources, such as transport, heating and industry, and can be classified as toxic, while TE occurring at higher concentrations in plant tissue, especially Zn and Mg, are micronutrients which are relatively easily taken up from the soil. These findings are in line with the work of Deljanin et al. (2016) who showed that TE emitted from anthropogenic sources (Al, V, Cr, Cu, Zn, As, Cd and Sb) are localised on the foliage surface, while micronutrients (Mn, Ni, Cu and Zn) accumulate in leaf tissue, but their uptake can be both by roots and foliar. In this study, root uptake could not be significant, because pH (> 7) of the soil in the study location does not favour TE uptake. The composition of TE in plants is indicative of their origin (Freer-Smith et al. 2005; Gandois and Probst 2012; Hovmand et al. 2009; Kocic et al. 2014); therefore, the higher concentration of Cr in plant tissues compared to PM deposited on foliage is surprising, especially if its low concentration in soil is taken into account. Cr, similar to Pb and Ni, is known as a good indicator of air pollution from traffic sources (Ny and Lee 2011), as it is related to vehicle emission from chromium trioxide in catalytic converters and from the rubber in car tires (Galvagno et al. 2002; Pastuszka et al. 2010). Cr distribution in plant tissues can be explained by the fact that Cr can enter plant leaves via foliar transfer and further be relocated to leaf cells (Shahid et al. 2017; Uzu et al. 2010). Furthermore, Gandois and Probst (2012) showed that some TE (e.g. Al, Mn, Co, Ni, and Zn) are labile and even if deposited as PM. They may migrate through the epicuticular wax to the internal part of plants.

The three tested species differed in terms of concentrations of TE in plant tissue, and the highest concentrations, except for Sb, were recorded in *C. betulus*. The differentiation between species was not surprising, since it was shown in earlier studies that there may be a more than 10-fold difference in TE accumulation between species, even when studied under the same pollution load conditions (Sæbø et al. 2012, 2015). It is interesting, however, that the highest TE concentrations inside the leaves were noted in deciduous *C. betulus*, not in conifers, especially not *P. nigra* which accumulated significantly higher amounts of PM. These results stand in contrast to Mori et al. (2015) and Przybysz et al. (2014b) who proved the high efficiency of conifers in TE accumulation. On the contrary, Reimann et al. (2007) showed that the coniferous spruce has low concentrations of most nutrients and some TE (Ba, Co, La, Na, Pb, Sb and Sr) in needles. This may demonstrate a good protective ability of the wax layer on the needles of conifers which are tolerant against pollutant deposition and translocation, or it may reflect a lower uptake of many TE in the foliage of plants that do not shed their leaves annually (Reimann et al. 2007). In the case of this work, both scenarios are possible and are supported by the thick wax layer protecting the *P. nigra* tissue against TE migration from needle surface into internal tissues. The concentration of TE in the needles of coniferous plants is also age dependent. Some TE of atmospheric origin (e.g. Cd, Cu and Pb) are mainly located in the wax of young needles, while in older needles, thus of the similar age to these examined in this work, partial assimilation and translocation to other organs as well as leaching from the needles by precipitation decrease TE concentrations (Gandois and Probst 2012).

In this study, we provide clear evidence that airborne PM is an important source of TE, including heavy metals, and that plants, if used properly, can serve as efficient green filters purifying ambient air from toxic TE. The TE were recorded in all PM size fractions accumulated on plant foliage and were rather equally distributed between _S_PM and _W_PM. On average, Zn, Ni, Pb and Cd were found in the highest concentrations in fine PM, while Sb in coarse PM. Large differences between species were found within individual TE, e.g. the highest concentration of Pb in *T. baccata* was recorded in coarse PM, while in *P. nigra* in fine PM. Only in the case of Sb the highest concentrations always found in coarse PM. According to Dubinskaya (1998) Cu, Cs, Zn, As, Cd and Pb are recorded in 75% portion of PM2 (smaller than 2 μm), while Cu, Mn and Fe are mainly attached with PM10 (smaller than 10 μm). Canepari et al. (2008) reported that elements of anthropogenic origin, i.e., vehicle emissions, non-tailpipe traffic sources, and railway emissions were found in the fine fraction (>50% of the total concentration of Pb and Cd being in the size fraction < 1), whereas Ba, Fe, Mg and Mn were mostly in the coarse fraction. Our results are only partly consistent with the above findings and rather indicate that TE are associated with PM of all size fractions and great differences might be observed between the studied plant species but probably also the location, the emission sources and the season of the year.

## Conclusions

In this study, PM and TE were accumulated on the foliage of three tested species. Most of accumulated PM belonged to the large fraction size (10–100 μm) and occurred on the surface of the foliage (_S_PM). The highest amounts of PM accumulated on the foliage of *P. nigra*, while TE on the leaves of *C. betulus*. The concentrations of four TE (Ni, Pb, Cd and Sb) were higher in PM accumulated on the foliage, while the highest concentrations of three others (Zn, Cr and Mg) were recorded in plant tissue. The TE were recorded in all PM size fractions and were equally distributed between surface PM and in-wax PM. These findings have implications for urban plantings in countries with a short vegetative season, where tolerant coniferous species and deciduous species which keep the last year’s foliage in winter should be included in urban forest plantings due to their efficiency in the removal of pollutants from the air.
